# Structural Analyses of CrtJ and Its B_12_-Binding Co-Regulators SAerR and LAerR from the Purple Photosynthetic Bacterium *Rhodobacter capsulatus*

**DOI:** 10.3390/microorganisms10050912

**Published:** 2022-04-27

**Authors:** Vladimira Dragnea, Giovanni Gonzalez-Gutierrez, Carl E. Bauer

**Affiliations:** Molecular and Cellular Biochemistry Department, Indiana University, Bloomington, IN 47405, USA; vdragnea@indiana.edu (V.D.); giovgonz@indiana.edu (G.G.-G.)

**Keywords:** PpsR ortholog, AerR photoreceptor, light regulation, photosynthesis gene regulators

## Abstract

Among purple photosynthetic bacteria, the transcription factor CrtJ is a major regulator of photosystem gene expression. Depending on growing conditions, CrtJ can function as an aerobic repressor or an anaerobic activator of photosystem genes. Recently, CrtJ’s activity was shown to be modulated by two size variants of a B_12_ binding co-regulator called SAerR and LAerR in *Rhodobacter capsulatus*. The short form, SAerR, promotes CrtJ repression, while the longer variant, LAerR, converts CrtJ into an activator. In this study, we solved the crystal structure of *R. capsulatus* SAerR at a 2.25 Å resolution. Hydroxycobalamin bound to SAerR is sandwiched between a 4-helix bundle cap, and a Rossman fold. This structure is similar to a AerR-like domain present in CarH from *Thermus termophilus*, which is a combined photoreceptor/transcription regulator. We also utilized AlphaFold software to predict structures for the LAerR, CrtJ, SAerR-CrtJ and LAerR-CrtJ co-complexes. These structures provide insights into the role of B_12_ and an LAerR N-terminal extension in regulating the activity of CrtJ.

## 1. Introduction

The presence of dioxygen has long been known to repress the synthesis of the structural and pigment components of the *Rhodobacter* photosystem [1]. Several well-characterized transcription factors, such as the RegA-RegB signaling cascade, FnrL, and CrtJ, are known to regulate the expression of photosynthesis and other genes in response to changes in cellular redox [1,2,3,4,5]. 

Orthologs of the transcription regulator CrtJ are present in many purple bacterial species, where they are responsible for controlling the expression genes involved in the synthesis of heme, BChl, carotenoids, and photosystem apoproteins [5,6,7,8,9]. CrtJ’s main action is to repress photosynthetic gene expression under aerobic conditions [5,6,7,8,9], although recent transcriptomic analysis showed that CrtJ also controls anaerobic gene expression [10,11]. CrtJ binds to promoters as a tetramer at the conserved palindrome sequence TGT-N12-ACA. Two copies of this palindrome are present as closely linked (typically separated by eight nucleotides) or as two distant sites that likely come together by DNA looping [12,13,14,15]. Until recently, it was presumed that the main control of CrtJ’s activity involved the oxidation/reduction of two conserved Cys, one of which is present in the DNA binding domain [16,17]. However, in vivo transcriptome analysis shows that CrtJ binds to many promoters under both aerobic and anaerobic conditions. Furthermore, CrtJ binding to target promoters often coincides with its regulatory partner AerR [10]. While AerR itself does not exhibit DNA binding activity, it is cotranscribed with CrtJ [18] and appears to be the main controller of CrtJ ability to either repress or activate gene expression [10,11,17]. 

**Figure 1:**
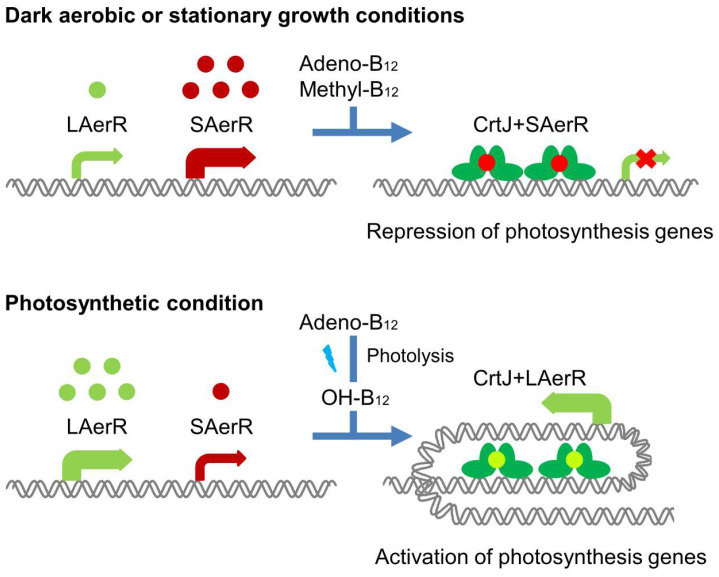
An updated model originally published in [11] that depicts the current understanding of the regulation of photosynthesis gene expression by CrtJ and its co-repressor SAerR (top) and its co-activator LAerR (bottom).

It has recently been shown that *Rba. capsulatus* synthesizes two isomeric forms of AerR, a short form called SAerR, and a longer variant called LAerR with a 40-amino acid extension at its amino terminus [11]. SAerR predominates in cells grown under dark aerobic conditions, as well as in cells that are in the stationary phase of growth. In contrast, the longer variant LAerR predominates in cells actively growing under anaerobic photosynthetic conditions (Figure 1). Yamamoto et al. [11] demonstrated that SAerR is generated from a second internal translation initiation site present within in the LAerR coding sequence. SAerR has been shown to direct CrtJ to function as an aerobic repressor at the puc (LHII), *bchEJGP, CrtA-bchDIO* and *crtB* operons [11]. Interestingly, the LAerR variant that predominates under photosynthetic conditions converts CrtJ into an activator of photosystem gene expression [11]. In vitro (DNase I protection) and in vivo (ChIP-exo) footprint studies on the *bchC* promoter show that CrtJ complexed with LAerR forms a greatly extended interaction with the *bchC* promoter that extends ~120 bases beyond which is protected by CrtJ alone [10]. The current model suggests that SAerR likely enhances CrtJ repression by increasing the binding of CrtJ to its recognition palindrome, while LAerR functions to induce an extended wrapped DNA complex around a CrtJ+LAerR co-complex that converts CrtJ into an activator (Figure 1) [11]. Unraveling details of the mechanism by which LAerR and SAerR can alter CrtJ DNA binding activity and CrtJ repressor versus activator functions is the goal of this study.

LAerR and SAerR also have very interesting novel functions as photoreceptors that uses B_12_ as a chromophore [11,17,19,20]. Studies have shown that LAerR tightly binds hydroxyl (OH-) cobalamin that is generated as a byproduct of light excitation of adeno or methyl-cobalamin. Cobalamin binding to LAerR involves the formation of a lower axial ligand to the corrin ring cobalt with His145 and the formation of an upper axial ligand to the same cobalt with His10 [17]. SAerR lacks the first 40 amino acids that are present in LAerR, so SAerR does not contain His10, which forms the upper axial ligand [11]. This alteration allows SAerR to exhibit less of a corrin ring selectivity, as this shorter variant binds adeno-, metyl, cyano- and hydroxyl-cobalamin irrespective of light exposure [11]. 

There are also interesting and informative variants in other species that exhibit similarities to the CrtJ-AerR regulatory mechanism. For example, *Thermus thermophilus* codes for a bacterial transcriptional regulator called CarH that has a B_12_-binding AerR domain linked to a DNA-binding regulator of carotenoid biosynthesis genes [21]. In CarH, photoexcitation of adenosyl-B_12_ leads to the dissociation of the upper liganded adenosyl group from B_12_, followed by the tight formation of a new upper ligand to a His in CarH [22,23]. CarH photochemistry is very similar to that observed with LAerR. Specifically, dark binding of adeno-B_12_ to CarH results in the formation of a CarH tetramer capable of binding and repressing carotenoid gene expression. Upon light excitation of the cobalamin ring, there is replacement of the upper adeno-Co ligand with a His that causes a conformational change in CarH which promotes dissociation into monomers incapable of binding DNA [22,23]. CarH’s linked AerR-DNA binding motif thus provides *T. thermophilus* with a mechanism to induce carotenoid gene synthesis when cells are exposed to blue light. Furthermore, a crystal structure of CarH has been solved in various states, dark, free, or bound to DNA and also when light-excited [22,23]. A fast spectroscopy study [22] also provided insights into light signaling by B_12_, which does not involve the formation of a radical pair, as is common in B_12_ photochemistry in solution or during enzymatic activity [24]. 

A second interesting variant occurs in *Rhodobacter sphaeroides*, which has a CrtJ ortholog called PpsR and an ortholog of AerR called PpaA [25]. The PpaA/AerR variant in this species has not been well studied beyond the observation that it also binds B_12_ in a light-dependent manner [19]. However, *Rba. sphaeroides* also codes for an AerR variant called AppA that has a flavin binding BLUF photoreceptor domain (blue light involving flavin) [26,27,28] linked to a AppA-like domain called the sensor containing heme instead of cobalamin (SCHIC) domain that binds heme instead of B_12_ [29,30]. Furthermore, AppA regulates PpsR activity in response to blue light absorption via FAD bound to the BLUF domain as well as heme availability sensed by the AppA-like domain [28,30,31]. As with CarH, there is a crystal structure of a PpsR deletion construct that lacks the DNA binding domain [32] as well as a crystal structure of the AppA FAD-binding BLUF domain [32,33], the SCHIC domain [30,32], and of a PpsR dimer bound to a monomer of AppA [32]. 

In this study, we provide a crystal structure of SAerR and models of LAerR and CrtJ that were constructed with Deep Mind AlphaFold software [34]. The LAerR and CrtJ *in-silco* models were validated by comparison with the known SAerR and PpsR structures. Docking of SAerR and LAerR to CrtJ as modeled on the solved PpsR2-AppA co-complex provides new structural insights as to the role of SAerR and LAerR in converting CrtJ from a repressor into an activator, respectively.

## 2. Materials and Methods

### 2.1. Protein Purification

We used previously described constructs pSUMO-SAerR and pSUMO-LAerR to express and purify both the short and long variants of AerR [11]. LAerR contains the full-length AerR sequence, while SAerR lacks forty N-terminal amino acids. For protein expression, the *E. coli* strain BL21(DE3) containing either pSUMO-SAerR or pSUMO-LAerR was grown on LB and kanamycin (25 µg/mL) at 37 °C to an optical density of A_600_ = 0.6. The flasks were then chilled to 16° C, and AerR expression was induced with the addition of IPTG to a final concentration of 50 µM with expression continued overnight. Cells were harvested by centrifugation at 10,000× *g* for 10 min with cell pellets stored at −80 °C until further use.

For AerR purification, cells were resuspended in buffer A: 20 mM Tris-HCl, pH 8.0, 0.3 M NaCl, 5 mM imidazole, and 10% glycerol to which 100 µM (final concentration) adenosyl cobalamin was added to the cell suspension. Cells were lysed with 3 passes through a French press with cell debris removed by centrifugation at 20,000× *g* for 10 min. The clarified supernatant was loaded into a superloop, briefly illuminated with strong white light (~1 min with Nikon High Intensity Illuminator NI-150 at maximum output). The sample was loaded onto the HisTrap FF column (ÄKTA FPLC) with unbound proteins removed by washing with 20 column volumes of 45 mM imidazole in buffer A. Elution of AerR was performed with a step up of 100% buffer B (same as buffer A but containing 500 mM imidazole). In this process, 3 mL of the eluted B_12_ containing the AerR pink sample was cleaved from the SUMO tag by addition of 2 mM DTT and 0.2 µM SUMO protease, and then incubated overnight at 4 °C. The AerR sample was then desalted on Econo-Pack 10DG desalting column (Bio-Rad, Hercules, CA, USA) in buffer A. To remove the cleaved SUMO tag, the desalted sample was then passed through 1 mL of His resin (Bio-Rad) that was equilibrated with Buffer A. The sample was then concentrated to ~1 ml using 10 K Amicon concentrators and loaded onto the Superose 12 size-exclusion column pre-equilibrated with 20 mM Tris 8.0 and 200 mM NaCl. The eluted AerR protein was then concentrated to 6 mg/mL for crystallization trials. 

For CrtJ purification, CrtJ was purified from a SUMO-CrtJ construct as described by Cheng et al. [35] using *E. coli* BL21DE3. After cleavage with Sumo protease, sample was applied directly on Superose 12 size-exclusion column as the SUMO tag (12 kDa) and CrtJ (>100 kDa) were easily size separated based on their different sizes. 

### 2.2. Absorption Spectroscopy 

UV-VIS spectra were recorded on Beckman DU-640 spectrophotometer.

### 2.3. DNase Footprint Analysis 

*bchC* promoter (325 bp) was amplified with 6-carboxyfluorescein phosphoramidate (FAM)-labeled primers as described previously [36]. We used 10 nM of the PCR amplified *bchC* promoter DNA segment in buffer 20 mM Tris –HCl, pH 8.0, 2 mM MgCl_2_, 0.5 mM CaCl_2_, 0.1 mg/mL BSA, and 5 uM protein (either purified CrtJ or sAerR). After 15 min of incubation, DNase I was added to the reaction to a 4 U/µL final concentration. After 15 min of digestion, the reaction was stopped by the addition of 0.5 M EDTA. The reactions were analyzed using GENEWIZ service (Washington, DC, USA).

### 2.4. Crystallization

Crystallization trials of SAerR at a protein concentration of 6 mg/mL used a hanging drop vapor diffusion method 1:1 *v/v* of protein to Hampton Research Index screen trial buffer. Protein crystallized as small diamond-shaped pink crystals that appeared within a few days in Hampton Research Index Screen N. 11. (3 M NaCl and 0.1 M Hepes 7.5). A subsequent additive screen (Hampton Research) yielded larger crystals grown with 1,6-hexandiol. The largest crystals were obtained in 3.2 M NaCl, 0.1 M Hepes pH 7.8 with 8% 1,6-hexandiol. Crystals were harvested directly from the hanging drop and flash frozen in liquid nitrogen. Diffraction data were collected at 100 K at Beamline station 4.2.2 at the Advanced Light Source (Berkeley National Laboratory, Berkeley CA, USA) and indexed, integrated, and scaled using XDS [37].

### 2.5. Structure Calculations

Two complete datasets collected at wavelengths of 1.00003 Å and 1.5998 Å were in space group P6122 at a 2.25 Å resolution (Supplementary Table S1). The phase estimation was performed using single anomalous diffraction (SAD). Initially, weak phases were estimated using Autosol and one Co site, which led to a figure of merit (FOM) of ~0.3 and an incomplete model containing only 26 well-placed residues of an α-helix and Rwork/Rfree of 0.4291/0.4965. This incomplete model was used to improve phase estimation by MR-SAD with a final FOM of ~0.6. This model gave rise only to Rwork/Rfree of 0.3846/0.4643, which was sufficient to perform molecular replacement with the native dataset. Successive cycles of automatic building in Autobuild (PHENIX) and manual building in Coot, as well as refinement (PHENIX Refine), led to a model missing only 8 residues (PDB code 7TE2). The final model showed a Rwork/Rfree of 0.2308/0.2590, which is approximately the 70th percentile compared to structures with a similar 2.25 Å resolution with excellent geometry and no Ramachandran outliers. All data collection statistics are shown in shown in Supplementary Table S1.

### 2.6. 3D Modeling Software

Alphafold CASP13 software [34] was used to model LAerR and CrtJ protein structures. Images were generated using PyMol.

## 3. Results

We initially set up hanging drop crystallization screens with both the short SAerR and long LAerR variants and a complex of these variants with CrtJ bound to DNA (26 or 27 bp oligomer of the *bchC* CrtJ binding sequence). LAerR and complexes with CrtJ/DNA/AerR did not crystallize in conditions that we tried. However, SAerR readily formed crystals under several conditions in Hampton Research Index Screen N.11. After optimization screening, we obtained well-diffracting pink diamond-shaped crystals that diffracted at a 2.25 Å resolution with Rwork/Rfree values of 0.2308/0.2590. Additional data collection and final refinement statistics are presented in Supplementary Table S1.

### 3.1. Structure of SAerR

**Figure 2:**
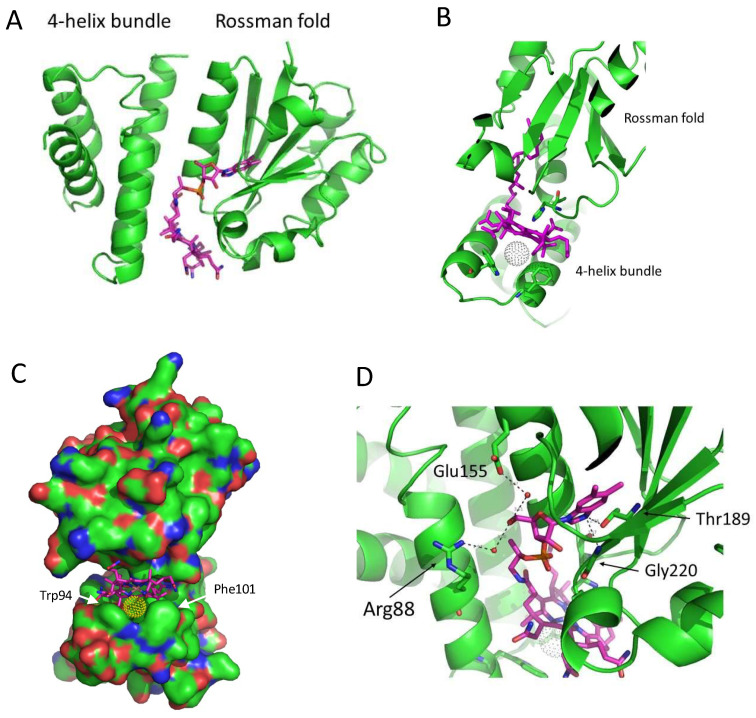
Ribbon representation of the SAerR crystal structure with bound cobalamin. (**A**) The flexible loop between the 4-helix bundle cap on the left and Rossman fold on the right. Missing in this structure is a flexible joining loop between these domains. (**B**) His145 forms the lower axial ligand while Cl (grey dotted circle) forms the upper axial ligand to cobalamin. Two aromatic residues Trp 94 and Phe101 in the 4-helix bundle also form a “cap” on the upper Cl ligand to cobalamin. (**C**) A space filling representation showing the cleft that holds cobalamin as well as the Cl atom (gold dotted circle) (**D**) H-bonding network with cobalamin. Red dots are water molecules. Note that the Cl atom has replaced the -OH upper ligand in the hydroxycobalamin attached to SAerR molecules with the 5-carbon sugar of cobalamin.

The solved crystal structure of SAerR containing bound cobalamin has a four-helix bundle cap followed by an α/β Rossman fold. A flexible loop between the four-helix bundle and the α/β Rossman fold is not resolved in the structure (Figure 2A). In general, this SAerR structure closely resembles other cobalamin binding domains whose structures have been solved, such as the methyl cobalamin-binding domain of methionine synthase MetH [38] and the B_12_-binding domain in the combined photoreceptor and transcriptional regulator CarH [23]. As shown in Figure 2A, B_12_ is nestled between the four-helix bundle cap on the left and the Rossman fold on the right. The corrin ring is anchored in place by His145 in the Rossman fold that functions as the lower ligand to the Co in cobalamin (Figure 2B). 

Pseudo-covalent attachment of B_12_ in AerR, as well as in CarH, involves light excitation of the adeno- or methyl-B_12_ cobalamin ring, which catalyzes the replacement of the upper Co axial ligands adeno or methyl group with another residue [17,20,23,24]. In CarH, and in LAerR, the upper adeno or methyl ligand is replaced with a histidine (His10 in AerR) [17,20,23,24]. In SAerR, His10 is not present, as this variant is 40 AA shorter at the amino terminus relative to LAerR [11]. In this case, the best fit for an upper axial ligand in the SAerR electron density map is an atom of Cl (dappled ball in Figure 2B,C). SAerR crystals were grown in a high concentration of NaCl, so Cl appears to have replaced the upper ligand –OH that was originally present in hydroxycobalamin that was light-attached to SAerR. Two additional Cl atoms were found in the structure, as well as a molecule of the additive 1,6- hexandiol that we used for the optimization of crystal growth. Analysis of the region near the upper Cl axial ligand shows that it is protected by two aromatic residues, Trp94 and Phe101, in SAerR (Figure 2B,C). Trp94 is also conserved in CarH but is replaced by Phe in MetH where the upper axial ligand to B_12_ Co is a methyl leaving group.

Several hydrogen bonds stabilize cobalamin in place by interacting with its long tail (Figure 2D). Specifically, there are H-bonds between nitrogen in the 5,6-dimethylbenzimidazole ring of cobalamin and highly conserved Gly220, and with a hydroxyl group of Thr189. Thr189 is replaced in other B_12_-binding domains such as MetH and CarH by Ser or Ala. Arg88 from the four-helix bundle and Glu155 from the Rossman fold also form a H-bond through two water molecules.

Interestingly, many residues forming an additional H-bond with B_12_ in the SAerR structure are not highly conserved. The few exceptions are the highly conserved residue Gly220 and the lower ligand of cobalamin His145 (marked by red boxes in Figure 2).

**Figure 3:**
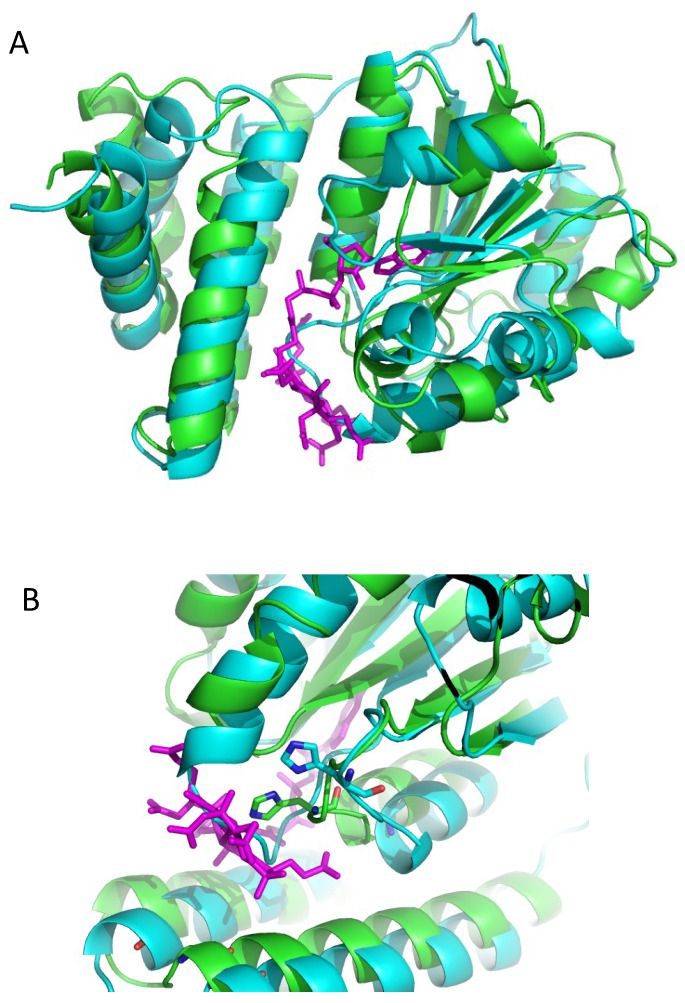
Structure of SAerR (green) containing cobalamin (purple) overlaying the SCHIC domain of AppA (blue). (**A**) A loop in the blue SCHIC domain which binds the heme occupies the place of cobalamin present in SAerR. (**B**) His284 in the AppA SCHIC domain is shifted by 2 positions (blue) from His145 in AerR that serves as lower axial ligand to heme and B_12_, respectively.

As discussed above, *Rba. sphaeroides* has a CrtJ orthlog called PpsR [8]. The activity of PpsR is regulated by PpaA, an AerR homolog [25], and by an additional photoreceptor/regulator called AppA [28,31]. AppA has a domain called the SCHIC domain that we previously crystallized [30]. This domain of AppA has structural features similar to that of B_12_-binding proteins, including AerR with the caveat that the AppA SCHIC domain binds heme instead of cobalamin [29,30]. Overlaying the SAerR and AppA SCHIC domain structures shows that it aligns closely with a RMSD value of 6.1Å. Inspection of this overlay shows several differences in the cobalamin binding region, the most obvious being an eight-amino acid loop in the SCHIC domain (residues Val329-Thr337) that likely hinders cobalamin binding (Figure 3A, blue). This loop connects the 3rd β-sheet and 3rd α-helix in the Rossman fold. In SAerR, this loop is much shorter (three residues: Val190-Met193), and in addition, the third β-sheet in SAerR is longer and tilted away from the B_12_ binding pocket providing more space for B_12_ in SAerR. His284 forms a Fe–heme ligand in SCHIC domain which corresponds to His145 in SAerR that forms the lower Co axial ligand (Figure 3B). However, the SCHIC His284 Fe ligand is shifted by two residues when aligned with other cobalamin binding domains. The “W94/F101 cap” protecting the B_12_ upper ligand in SAerR is also replaced in AppA SCHIC domain by L239 and R246, respectively. Collectively, these structural alterations between SAerR and the SCHIC domain of AppA appears to be responsible for the observed alteration of tetrapyrrole selectively between these two different photoreceptors. 

### 3.2. Alphafold 3D Modeling of LAerR 

**Figure 4:**
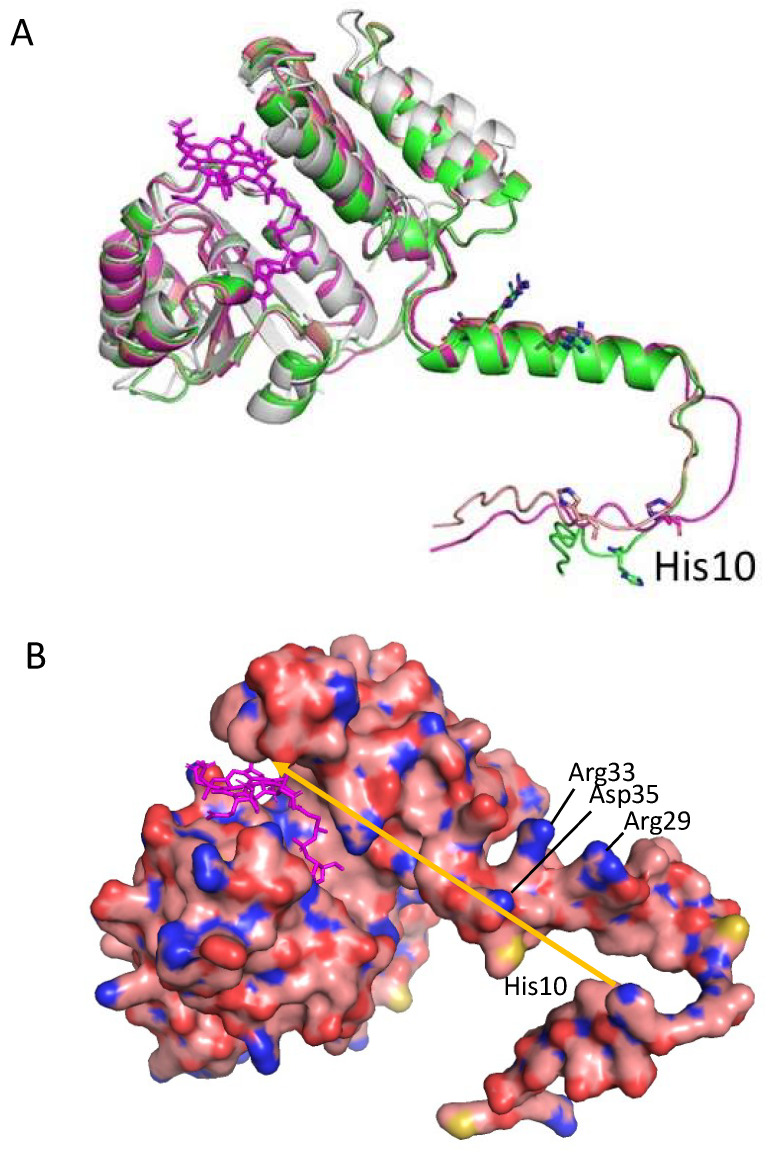
Crystal structure of SAerR (magenta) overlaid with the three best Alphafold predictions of LAerR. (**A**) The N-terminal 40 residues that are missing in SAerR are modeled in LAerR into an α1-helix preceded by a flexible tail that contains the upper Co ligand His10 shown as sticks. (**B**) Surface charges of LAerR with B_12_ (magenta; as positioned in SAerR). Oxygen atoms are red, nitrogen atoms are blue, and sulfur atoms are mustard yellow. The orange arrow shows the predicted movement of His10 that must occur to form the upper axial ligand to Co upon light excitation.

We were not able to obtain crystals of LAerR, as this longer variant is much less soluble and more heterogeneous than SAerR, as indicated by dynamic light-scattering. However, the modeling software AlphaFold does generate predictive 3D structures of proteins from a primary sequence with high confidence [34]. Using AlphaFold, we generated several models of LAerR 3D structures with the three best superimposed over each other in Figure 4A. 

These predictive structures of LAerR contains a 40 amino terminal longer section as compared to our solved crystal structure of SAerR. As shown in Figure 4A, the LAerR model structures are highly superimposed over the solved SAerR structure as represented in magenta. The RMSD score between the same residues in the SAerR structure to the same residues in the LAerR structure is 2.8, which indicates an excellent model fit. The N-terminal’s 40 amino acids in LAreR that are not present in SAerR are modelled into a long flexible tail (residues 1–18) followed by a long α-helix denoted as α1-helix (Phe19-Asn36). Mutational analysis of LAerR has shown that light excitation of adeno- or methyl-cobalamine allows His10 to form an upper ligand with the Co [20]. However, in this dark *i*n silico model of LAerR (where no B_12_ would be attached), we see that His10 in the flexible amino-terminal tail segment is at a considerable distance from where an upper axial ligand could be formed with cobalamin (Figure 4A,B). One clue for how His10 forms the upper Co axial ligand can be derived from the crystal structure of CarH, which has a similar B_12_ binding domain. The crystal structure of dark CarH shows that the lower ligand is also a His and that the upper ligand remains in the adeno group [22,23]. However, after light excitation of the corrin ring, the upper adeno axial ligand is released and replaced with a His, not unlike what occurs with LAerR. Furthermore, this light-mediated change in the upper axial ligand causes an ~8 Å movement of the 4-helix bundle cap, which then disrupts tetramerization and DNA binding of CarH [23]. We suspect that a similar movement of the same helix bundle cap occurs in LAerR that would allow His10 to form an axial ligand upon light excitation of B_12_. Finally, there is a deep groove on one side of LAerR, which could potentially be involved in the docking of the α1 N-terminal helix once there is light-driven formation of a His10 Co axial ligand (Figure 4B). 

### 3.3. Alphafold 3D Modeling of CrtJ 

**Figure 5:**
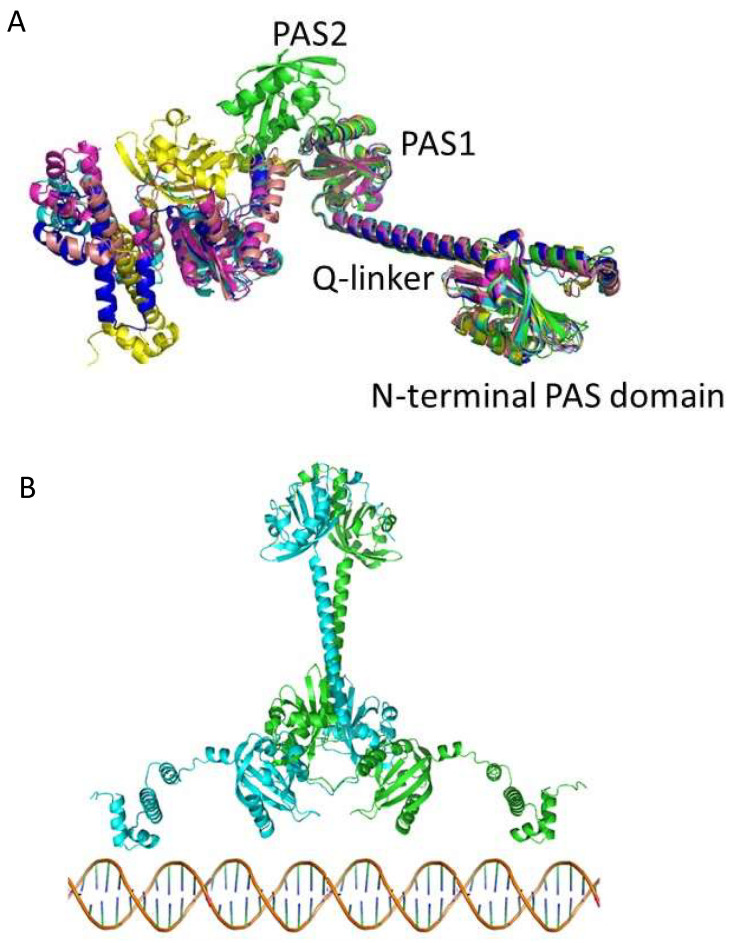
Crystal structure of PpsR from *Rba. sphaeroides* (in green) overlaid on the five best AlphaFold models of *Rba. capsulatus* CrtJ. (**A**) The crystallized portion of PpsR (right side of the figure) aligns very well with predicted CrtJ models, especially the N-terminal PAS domain, the long α-helix (Q linker) and the first PAS domain. The second PAS domain in PpsR is positioned differently from all of the CrtJ models. The models of the HTH DNA binding domain which is attached via a flexible region are also in variable positions. (**B**) Best CrtJ dimer model based on the *Rba. sphaeroides* PpsR dimer crystal structure (4HH2.pdb) CrtJ residues up to amino acid 261 are very well aligned with the PpsR crystal structure containing the N-terminal PAS domain, Q-linker a-helix and PAS1 with best RMSD = 5.5 Å at Cα atoms for CrtJ model in dark blue.

We also used AlphaFold to generate a predictive structure of CrtJ. CrtJ is a homolog of transcription regulator PpsR from *Rba. sphaeroides* that was previously crystallized without HTH DNA binding domain (PDB 4HH2.pdb) [32]. In Figure 5A, we show the five best in silico models of CrtJ aligned with the PpsR HTH crystal structure shown in green. 

The last 200 residues of CrtJ containing PAS2 and HTH vary from PpsR and also among the five models. This portion is likely very flexible, preventing the crystallization of full-length CrtJ. In Figure 5B, a model is shown of a CrtJ dimer as based on the PpsR structure [32]. 

### 3.4. A Model of a 2CrtJ-AerR Regulatory Complex 

**Figure 6:**
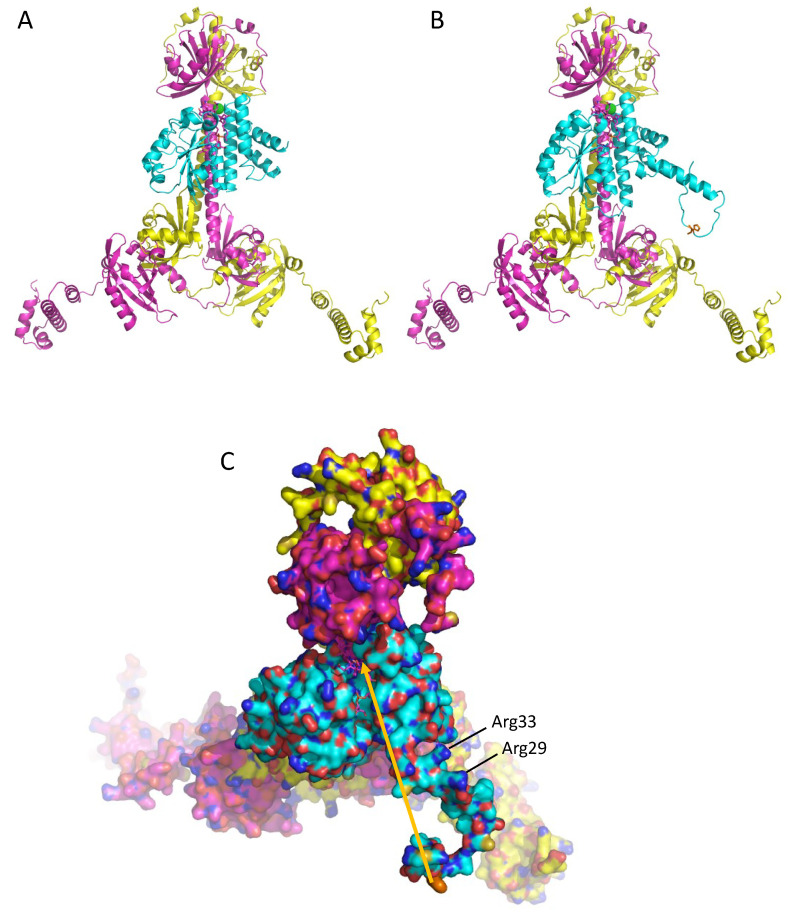
Model of SAerR and LAerR interactions with a CrtJ dimer. (**A**) Ribbon model of SAerR (blue) bound to the Q-linker helix region of a CrtJ dimer (magenta and yellow). (**B**) Ribbon model of LAerR (blue) bound to the Q-linker helix region of a CrtJ dimer (magenta and yellow. (**C**) Top orientation of a space filling model of LAerR (light blue) interacting with the Q-linker helix region of a CrtJ dimer (magenta and yellow). Oxygen atoms are red, nitrogen atoms are blue, and sulfur atoms are mustard yellow. The arrow shows where H10 must move upon light excitation. Positively charged Arg33 and Arg29 are also highlighted.

Finally, Winkler et al. [32] also obtained a crystal structure of AppA bound to a PpsR dimer (PDB 4HH3.pdb). In that structure, the SCHIC domain, which is structurally similar to AerR (Figure 3), interacts with the long Q-linker helix that separates the N-terminal PAS domain from the PAS1 domain. Using this structure as a guide, we constructed models of SAerR and LAerR bound to a CrtJ dimer at a similar location as the SCHIC domain interaction with PpsR (Figure 6A,B). Regarding this potential site of interaction, there are six residues (Gln133, Leu136, Gln140, Glu144, Tyr147 and Arg151) in the CrtJ Q-linker region that are universally conserved in all known CrtJ orthologs that face and align with the fourth helix of the four-helix bundle of AerR/SCHIC. There are also three residues in this AerR/SCHIC interacting helix that could form potential bonds with conserved residues in the Q-linker helix in CrtJ. These potential hydrogen bond interactions are: AerR Thr105 potentially H-bonding with Gln133 of CrtJ, AerR Ser109 potentially H-binding with Gln140 of CrtJ and AerR Gln112 potentially H-binding with Glu144 of CrtJ.

As discussed in the Introduction, SAerR converts CrtJ into a repressor while LAerR converts CrtJ into an activator [11]. One clue for how this may occur is the results of extensive in vitro and in vivo footprint assays which show that CrtJ alone, or when complexed with SAerR, exhibit a small ~44 base footprint at the *bchC* promoter region that contains two CrtJ recognition palindromes (one spanning the −10 and the other the −35 promoter region) [10,11]. This is contrasted by in vitro and in vivo footprints of CrtJ complexed with LAerR, which show that LAerR-CrtJ binding to the *bchC* promoter extends an additional ~120 nucleotides beyond the CrtJ recognition palindrome [10]. This result has given rise to a model where DNA is wrapped around an LAerR-CrtJ complex that then promotes the activation of transcription [11].

The main structural difference between the SAerR and LAerR variants is a 40-amino acid extension at the amino terminus of LAerR that contains a flexible region with His10 that forms an upper axial ligand to light-excited B_12_ and the 17-residue α1-helix (Phe19-Asn36) [11]. These two structural features in LAerR must therefore be responsible for altering CrtJ activity into an activator. Note that the AlphaFold model of LAerR does not contain B_12_, so the flexible amino-terminal region containing His10 is not positioned at the B_12_ binding pocket located on the narrow side of the Rossman fold. However, genetic evidence clearly demonstrates that His10 does form this upper axial ligand to B_12_ when the corrin ring is light-excited [17], so this region along with the α1-helix must move in light-excited LAerR to a position near the Rossman fold. There is also a flexible strand between the α1-helix and the four-helix cap that should allow α1-helix to rotate and move toward the Rossman fold upon the formation of a His10-Co axial ligand (Figure 4A). Furthermore, inspection of surface charges on the α1-helix shows that one side is composed of hydrophobic residues, while the other side has several positively charged Arg residues (Arg29, Arg33, Arg34, Asp36) (Figure 4B and Figure 6C). We propose that when His10 forms an axial ligand to the cobalamin Co, then the hydrophobic side of the α1-helix is likely positioned along a groove between the Rossman fold and the alpha cap to position its positively charged Arg residues in a way that allows an extended interaction of a looped region of the DNA helix with LAerR.

## 4. Discussion

In this work, we have solved a 2.25 Å resolution crystal structure of the short SAerR variant of AerR and used AlphaFold AI software [34] to obtain a predicted structure of the long variant LAerR. These two structures have a very high degree of similarity in regions of sequence conservation, specifically for the four-helix cap and Rossman fold that together contain a B_12_-binding domain. Both AerR variant structures also show excellent similarities to the SCHIC domain crystal structure from AppA that is known to bind heme instead of B_12_ [29,30,32]. These structural similarities provide validity to the LAerR structural model as generated by AlphaFold. Likewise, AlphaFold also generated a structure of CrtJ that is strikingly similar to the known crystal structure of its *Rba. sphaeroides* ortholog PpsR [32], again showing a high degree of confidence on the CrtJ structure provided by AlphaFold.

With these structures, we obtain new insights into the mechanism by which SAerR functions as an anaerobic CrtJ co-repressor while LAerR functions as a photosynthetic (light anaerobic) CrtJ co-activator [11]. For example, the role of light excitation of B_12_ in LAerR appears to be as a catalyst to promote the formation of a B_12_ upper ligand with His10. This event should pin the α1 helix to a specific position on the LAerR surface such that it promotes an additional positive charged region that can interact with DNA. Note that movement of the α1 helix would be a light-dependent event, which coincides with the role of LAerR to stimulate gene expression under photosynthetically logarithmic growth conditions [11]. The role of SAerR is to function as a dark/stationary phase co-repressor, so it likely that the binding of SAerR to CrtJ (and potentially also the binding of LAerR to CrtJ) likely helps to stabilize the dimerization of two CrtJ subunits, thereby increasing the binding affinity of this co-complex to target sequences. These are testable models that can be validated by future mutational analyses of critical protein–protein and protein–DNA interacting residues that are highlighted by this study.

Addressing the previously described regulatory role of the redox-active Cys in CrtJ was not a goal of this study. However, it is likely that these two AerR variants function as separate light-responding regulators that overlay their regulatory role over that of direct redox sensing by CrtJ. Specifically, prior studies have shown that a Cys in the HTH motif (Cys 420) can form a sulfenic acid modification in vivo when cells are exposed to oxygen [16,35]. Moreover, a Cys420 to Ala mutation leads to a ~60-fold reduction in DNA binding activity, while a Cys to Ser substitution at position 420 that mimics a cysteine sulfenic acid results in approximately a four-fold increase in DNA binding activity [35]. This Cys is located in the cluster of DNA recognition helices located at the carboxyl end of CrtJ. Additional structural analysis of CrtJ bound to DNA in oxidized and reduced forms both with and without SAerR and LAerR will be needed to obtain a unifying model of light and redox control of CrtJ repression and activation.

## Data Availability

X-ray crystallographic data have been deposited in the Protein Data Bank under accession code PDB code 7TE2.

## Supplementary Materials

The following supporting information can be downloaded at: https://www.mdpi.com/article/10.3390/microorganisms10050912/s1, Table S1: Data-collection and refinement statistics.
