# Supplementary material for: Structural Analyses of CrtJ and Its B12-Binding Co-Regulators SAerR and LAerR from the Purple Photosynthetic Bacterium Rhodobacter capsulatus
Source: Microorganisms. 2022 Apr 27;10(5):912. doi: 10.3390/microorganisms10050912 (PMC9144470; doi:10.3390/microorganisms10050912)
Supplement: Supplementary file 1 [file microorganisms-10-00912-s001.zip › microorganisms-1695641-supplementary.pdf]

Supplemental Table S1. Data-collection and refinement statistics

	SAerR	SAerR
Data collection		
Wavelength (Å)	1.00003	1.5998
Space group	P 61 2 2	P 61 2 2
Cell dimensions		
a, b, c (Å)		
α, β, γ (°)	90.00 90.00 120.00	90.00 90.00 120.00
Resolution (Å)	47.32 – 2.25	47.57 – 2.75
R_{merge}	0.104 (1.804)	0.162 (2.410)
R_{meas}	0.107 (1.861)	0.164 (2.444)
R_{pim}	0.024 (0.451)	0.026 (0.402)
Total reflections	217102 (16919)	246389 (32385)
No. unique reflections	11257 (1005)	6369 (891)
CC1/2	0.999 (0.687)	0.999 (0.831)
I/σ(I)	20.6 (1.7)	22.8 (1.8)
Completeness (%)	100.0 (100.0	100.0 (100.0)
Multiplicity	19.3 (16.8)	38.7 (36.3)
Refinement		
Resolution (Å)	47.32 – 2.25 (2.33 – 2.25)	
No. unique reflections	11189 (1083)	
R_{work}	0.2308 (0.3106)	
R_{free}	0.2599 (0.3307)	
R.m.s.d values		
Bond lengths (Å)	0.007	
Bond angles (°)	1.40	
No. atoms		
Protein	11572	
Ligand/ions	102	
Solvent	77	
B-factors ($Å^2$)		
Protein	30.11	
ligand/ions	27.60	
Solvent	30.50	
Ramachandran plot		
Favored (%)	98.5	
Allowed (%)	1.5	
Outliers (%)	0.0	
Rotamer outliers (%)	0.0	
Clashscore	6.14	
PDB code		

*Highest-resolution shell values are shown in parentheses.
